# Predicting the Early-Age Time-Dependent Behaviors of a Prestressed Concrete Beam by Using Physics-Informed Neural Network

**DOI:** 10.3390/s23146649

**Published:** 2023-07-24

**Authors:** Hyun-Woo Park, Jin-Ho Hwang

**Affiliations:** Department of ICT Integrated Safe Ocean Smart Cities, Dong-A University, 37 Nakdong-Daero 550beon-gil, Saha-gu, Busan 49315, Republic of Korea; hwangjinho@donga.ac.kr

**Keywords:** physics-informed neural network (PINN), prestressed concrete (PSC) beam, optimal hyperparameter combination, concrete creep, concrete shrinkage, tendon relaxation, early-age time-dependent behaviors, prestress loss

## Abstract

This paper proposes a physics-informed neural network (PINN) for predicting the early-age time-dependent behaviors of prestressed concrete beams. The PINN utilizes deep neural networks to learn the time-dependent coupling among the effective prestress force and the several factors that affect the time-dependent behavior of the beam, such as concrete creep and shrinkage, tendon relaxation, and changes in concrete elastic modulus. Unlike traditional numerical algorithms such as the finite difference method, the PINN directly solves the integro-differential equation without the need for discretization, offering an efficient and accurate solution. Considering the trade-off between solution accuracy and the computing cost, optimal hyperparameter combinations are determined for the PINN. The proposed PINN is verified through the comparison to the numerical results from the finite difference method for two representative cross sections of PSC beams.

## 1. Introduction

Most machine learning-based approaches for predicting the mechanical behaviors of structural systems require a huge amount of high-quality data for supervised learning. In many real-world engineering problems, such vast amounts of data are often unattainable on site, and limitations in data accessibility pose challenges for machine learning based on field data. To overcome these limitations, the physics-informed neural network (PINN) has gained attention for its capability of incorporating fundamental physical (or mechanical) knowledge, presented in the form of differential or integral equations, into machine learning [1,2,3,4,5,6,7,8].

The PINN addresses data limitation challenges by excelling in solving problems with uncertain or missing information [9] and holds the potential to significantly reduce computational costs [10]. Additionally, the PINN can be applied to problems in arbitrary domains and can scale to high-dimensional systems. In numerous scientific and engineering fields, the PINN is gaining increasing popularity today including material [11,12,13], nonlinear diffusivity [14], fluid mechanics [15,16,17,18], bioengineering [19], solid mechanics [20,21], geophysics [22], thermophysics [23], machine fault diagnosis [24], and scientific computations [25,26]. Furthermore, the PINN has been expanded to tackle integro-differential equations (IDEs) [27], fractional differential equations (FDEs) [28], stochastic differential equations (SDEs) [29,30,31,32], and shown to estimate the generalization error [33,34,35].

The PINN utilizes a feed-forward architecture combined with automated differentiation in artificial neural networks to train models that satisfy the governing equations, enabling the construction of physical prediction models with far fewer field data than traditional data-driven neural network techniques [36]. The PINN estimates the parameters that make up the neural network by optimizing an objective function composed of errors related to the governing equations and boundary (or initial) conditions. In this process, it is necessary to calculate the derivative values for the unknowns included in the governing equations or boundary conditions, and the PINN adopts automatic differentiation techniques instead of numerical differentiation [37]. This advantage allows for the direct handling of any form of differential operators for unknowns included in the governing equations. By using PINNs, it is possible to address the missing data and measurement errors in actual physics that could not be resolved with conventional approaches.

PINNs demonstrate constraints when handling issues that evolve sharp gradients or involve interconnected PDEs [38]. As a consequence, traditional PINNs have been improved by employing domain decomposition methods [39,40,41,42]. A generalized space-time domain decomposition framework for PINNs (XPINN) was proposed to solve any differential equations on arbitrary complex geometry domains [39,40]. The XPINN easily lends itself to space-time parallelization, thereby reducing training costs more effectively. Augmented PINN (APINN) was proposed to further improve the XPINN as well as vanilla PINNs by adopting soft and trainable domain decomposition and flexible parameter sharing [41]. The domain decomposition methods can reduce the complexity of PINNs by dividing the problem into smaller, more manageable subproblems. They also can improve the stability of PINNs by reducing the risk of overfitting since the subproblems are more independent, which can help to prevent the model from learning spurious correlations.

The incorporation of adaptive activation functions into traditional PINNs has emerged as a compelling alternative [43,44,45,46]. Adaptive activation functions can help PINNs to converge more quickly and reliably because they can adapt to the specific problem at hand, which can help to avoid getting stuck in local minima. They also can help PINNs to generalize better to unseen data since they can learn to represent the underlying physical laws more accurately improving the robustness of the model. Lastly, they can be less sensitive to hyperparameters than conventional activation functions. This is because they can adapt to the specific problem at hand, which can help to improve the stability of the training process.

In the civil engineering industry, prestressed concrete (PSC) beams are increasingly used in construction due to their advantages over reinforced concrete (RC) beams in controlling cracking and minimizing long-term deflections [47]. To design safe and reliable PSC beams, however, it is crucial to properly predict prestress losses, particularly in high-strength concrete, at an early age [48,49,50]. There have been numerous attempts to investigate the time-dependent behavior of PSC structures, including the effects of concrete creep and shrinkage, tendon relaxation, and changes in the concrete elastic modulus [51,52,53,54,55,56]. Most of these studies employ numerical models such as finite element and finite difference methods to predict the long-term behavior of prestressed concrete structures [57,58,59,60]. They are computationally intensive and require significant computational resources.

This paper presents a PINN-based approach for predicting the early-age time-dependent behaviors of a PSC beam considering interaction among concrete creep and shrinkage, tendon relaxation, and the changes of concrete elastic modulus. We introduce a novel closed-form integro-differential governing equation, formulated in terms of effective prestress force, to predict the early-age time-dependent behaviors of PSC beams. Contrary to the traditional finite element method, the PINN directly solves the integro-differential equation without requiring discretization. There exists a trade-off between solution accuracy and the computing time when solving the integro-differential governing equation through PINN. Balancing the importance of the solution accuracy and the computing cost, optimal hyperparameter combinations are determined for the PINN.

The paper is organized as follows. Section 2.1 presents eight dimensionless governing equations for the time-dependent behaviors of a PSC beam. Section 2.2 describes the derivation of the closed-form integro-differential equation in terms of dimensionless effective prestress force. Section 2.3 presents determining optimal hyperparameter combinations for the PINN to solve the integro-differential equation. Section 3.1 presents the results of optimal hyperparameter combinations for the PINN and a comparison with the finite difference method for PSC beams with rectangular and I-shaped cross sections. Finally, Section 4 summarizes the findings and their implications for the proposed PINN approach.

## 2. Materials and Methods

### 2.1. Governing Equations

#### 2.1.1. Problem Definition and Assumptions

**Figure 1:**
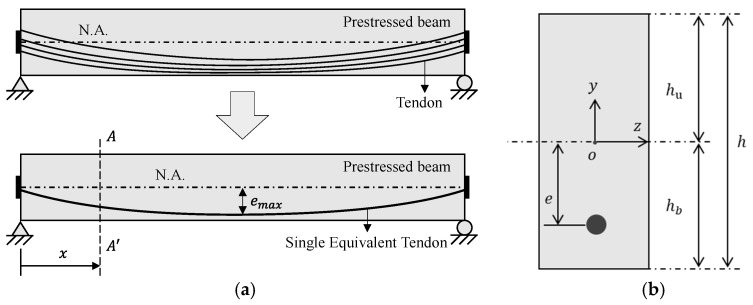
Simplified beam model for a PSC beam: (**a**) PSC beam model with a single equivalent tendon; (**b**) cross section at $AA^{\prime }$.

Figure 1 presents a simplified PSC beam model to analyze the early-age time-dependent behaviors of a precast prestressed concrete beam. The period of interest for the analysis spans approximately one year beginning from the initial prestress transfer to the installation of the beam at the intended target construction site. The assumptions for the time-dependent analysis of this study can be described as follows:

(i) Multiple tendons of the PSC beam can be substituted with a single equivalent tendon as shown in Figure 1a;

(ii) The tendon is perfectly bonded with concrete; thus, section analysis for equilibrium between the tendon and the concrete is possible;

(iii) The beam model follows the Euler–Bernoulli beam theory;

(vi) The time-dependent behaviors of concrete include the change of elastic modulus, creep, and shrinkage;

(v) The creep of concrete follows the standard linear model of viscoelasticity;

(vi) The relaxation of the tendon is assumed to be intrinsic;

(vii) The shrinkage of concrete occurs uniformly throughout the gross section of concrete;

(viii) The net strain of concrete consists of elastic strain, creep strain, and shrinkage strain;

(ix) The self-weight of the PSC beam is introduced at the moment of initial prestress transfer.

#### 2.1.2. Governing Equations for Time-Dependent Behaviors of a PSC Beam

In the simplified model for a PSC beam in Figure 1, a time-dependent analysis should be conducted at each section of interest to satisfy the equilibrium between the concrete and the tendon. For the simplicity of description, the spatial variable x corresponding to section $AA^{\prime }$ in Figure 1 is omitted in all field variables hereafter.

The governing equations for time-dependent behaviors can be expressed as follows:

(i) Axial-bending stress equation of concrete

(1) $$\sigma \left(y,t\right)=-P\left(t\right)\left(\frac{1}{A_{c}}-\frac{e}{I_{c}}y\right)-\frac{M_{d}}{I_{c}}y$$

where t and $M_{d}$ denote the time beginning from the initial prestress transfer and the bending moment induced by the self-weight of the beam, respectively.

(ii) Constitutive equation of elastic strain of concrete [61]

(2) $$\begin{array}{cc} \varepsilon_{\mathrm{el}}\left(y,t\right)=\frac{\sigma \left(y,t\right)}{E_{c}\left(t\right)} & =\Phi_{\mathrm{el}}\left(t\right)\frac{\sigma \left(y,t\right)}{E_{c0}}\\ & =-\Phi_{\mathrm{el}}\left(t\right)P\left(t\right)\left(\frac{1}{E_{c0}A_{c}}-\frac{e}{E_{c0}I_{c}}y\right)-\Phi_{\mathrm{el}}\left(t\right)\frac{M_{d}}{E_{c0}I_{c}}y \end{array}$$

where $E_{c0}$ is the elastic modulus of concrete at t=0 and $\Phi_{\mathrm{el}}\left(t\right)=\frac{E_{c0}}{E_{c}\left(t\right)}$.

(iii) Constitutive equation of creep strain of concrete [62]

(3) $$\varepsilon_{\mathrm{cr}}\left(y,t\right)=\int_{0}^{t}\frac{{\tilde{\Phi }}_{\mathrm{cr}}\left(t,\tau \right)}{E_{\mathrm{ci}}}\frac{\partial \sigma \left(y,\tau \right)}{\partial \tau }d\tau +\frac{{\tilde{\Phi }}_{\mathrm{cr}}\left(t,0\right)}{E_{\mathrm{ci}}}\sigma \left(y,0\right)$$

where $E_{\mathrm{ci}}$ is the elastic modulus of concrete at the age of 28 days while ${\tilde{\Phi }}_{\mathrm{cr}}\left(t,\tau \right)$ is the creep coefficient at time t for the unit strain of which the corresponding stress is $E_{\mathrm{ci}}$ applied at time τ.

(iv) Net strain of concrete

(4) $$\varepsilon_{\mathrm{net}}\left(y,t\right)=\varepsilon_{\mathrm{el}}\left(y,t\right)+\varepsilon_{\mathrm{cr}}\left(y,t\right)+\varepsilon_{\mathrm{sh}}\left(t\right).$$

(v) Constitutive equation of elastic strain of the tendon

(5) $$\varepsilon_{p\_\mathrm{el}}\left(t\right)=\frac{P\left(t\right)}{E_{p}A_{p}}+\varepsilon_{p\_d}\left(t\right)$$

where $\varepsilon_{p\_d}\left(t\right)=\Phi_{\mathrm{el}}\left(t\right)\frac{M_{d}}{E_{c0}I_{c}}e$ representing the elastic strain of concrete at the location of the tendon due to the self-weight of the beam at time t.

(vi) Constitutive equation of relaxed strain of the tendon

(6) $$\varepsilon_{p\_\mathrm{re}}\left(t\right)=\Phi_{\mathrm{re}}\left(t,0\right)\varepsilon_{p\_\mathrm{el}}\left(0\right).$$

(vii) Net strain of the tendon

(7) $$\varepsilon_{p}\left(t\right)=\varepsilon_{p\_\mathrm{el}}\left(t\right)+\varepsilon_{p\_\mathrm{re}}\left(t\right).$$

(viii) Compatibility between the concrete and tendon at y=−e

(8) $$\frac{\partial \varepsilon_{p}\left(t\right)}{\partial t}=\frac{\partial \varepsilon_{\mathrm{net}}\left(-e,t\right)}{\partial t}.$$

#### 2.1.3. Dimensionless Governing Equations

For the simplicity of formulation in Section 2.2, governing equations from Equation (1) to Equation (8) in Section 2.1.2 are rewritten by introducing dimensionless variables normalized by respective mechanical quantities. Force, strain, and stress variables are normalized by $P_{i}$, $\frac{P_{i}}{E_{p}A_{p}}$ and $E_{c0}\left(\frac{P_{i}}{E_{p}A_{p}}\right)$, respectively. The normalized variables will be denoted by using 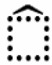 otherwise mentioned hereafter. For example, substituting $\sigma \left(y,t\right)=E_{c0}\left(\frac{P_{i}}{E_{p}A_{p}}\right)\hat{\sigma }\left(y,t\right)$ and $P\left(t\right)=P_{i}\hat{P}\left(t\right)$ into Equation (1) results in:

(i) Dimensionless axial-bending stress equation of concrete

(9) $$\hat{\sigma }\left(y,t\right)=-\gamma \left(y\right)\hat{P}\left(t\right)-{\hat{\sigma }}_{p\_d}\frac{y}{e}$$

where $\gamma \left(y\right)=n\left(0\right)A_{p}\left(\frac{1}{A_{c}}-\frac{e}{I_{c}}y\right)$ and ${\hat{\sigma }}_{p\_d}=\left(\frac{M_{d}}{I_{c}}e\right)/\left(\frac{E_{c0}P_{i}}{E_{p}A_{p}}\right)$ implying the normalized stress at the location of the tendon due to the self-weight of the beam.

In a similar fashion, Equations (2)–(8) can be rewritten as follows:

(ii) Dimensionless constitutive equation of elastic strain of concrete

(10) $${\hat{\varepsilon }}_{\mathrm{el}}\left(y,t\right)=-\Phi_{\mathrm{el}}\left(t\right)\gamma \left(y\right)\hat{P}\left(t\right)-{\hat{\varepsilon }}_{p\_d}\left(t\right)\frac{y}{e}$$

where ${\hat{\varepsilon }}_{p\_d}\left(t\right)=\varepsilon_{p\_d}\left(t\right)/\left(\frac{P_{i}}{E_{p}A_{p}}\right)$.

(iii) Dimensionless constitutive equation of creep strain of concrete

(11) $${\hat{\varepsilon }}_{\mathrm{cr}}\left(y,t\right)=\int_{0}^{t}\Phi_{\mathrm{cr}}\left(t,\tau \right)\frac{\partial \hat{\sigma }\left(y,\tau \right)}{\partial \tau }d\tau +\Phi_{\mathrm{cr}}\left(t,0\right)\hat{\sigma }\left(y,0\right)$$

where $\Phi_{\mathrm{cr}}\left(t,\tau \right)=\frac{E_{c0}}{E_{\mathrm{ci}}}{\tilde{\Phi }}_{\mathrm{cr}}\left(t,\tau \right)$ implying the creep coefficient at time t for the unit strain of which the corresponding stress is $E_{c0}$ applied at time τ.

(iv) Dimensionless net strain of concrete

(12) $${\hat{\varepsilon }}_{\mathrm{net}}\left(y,t\right)={\hat{\varepsilon }}_{\mathrm{el}}\left(y,t\right)+{\hat{\varepsilon }}_{\mathrm{cr}}\left(y,t\right)+{\hat{\varepsilon }}_{\mathrm{sh}}\left(t\right).$$

(v) Dimensionless constitutive equation of elastic strain of the tendon

(13) $${\hat{\varepsilon }}_{p\_\mathrm{el}}\left(t\right)=\hat{P}\left(t\right)+{\hat{\varepsilon }}_{p\_d}\left(t\right).$$

(vi) Dimensionless constitutive equation of relaxed strain of the tendon

(14) $${\hat{\varepsilon }}_{p_{\mathrm{re}}}\left(t\right)=\Phi_{\mathrm{re}}\left(t,0\right){\hat{\varepsilon }}_{p_{\mathrm{el}}}\left(0\right).$$

(vii) Dimensionless net strain of the tendon

(15) $${\hat{\varepsilon }}_{p}\left(t\right)={\hat{\varepsilon }}_{p\_\mathrm{el}}\left(t\right)+{\hat{\varepsilon }}_{p\_\mathrm{re}}\left(t\right).$$

(viii) Dimensionless compatibility between the concrete and tendon at y=−e

(16) $$\frac{\partial {\hat{\varepsilon }}_{p}\left(t\right)}{\partial t}=\frac{\partial {\hat{\varepsilon }}_{\mathrm{net}}\left(-e,t\right)}{\partial t}.$$

Because there are eight unknown dimensionless variables $\hat{\sigma }\left(y,t\right)$, $\hat{P}\left(t\right)$, ${\hat{\varepsilon }}_{\mathrm{el}}\left(y,t\right)$, ${\hat{\varepsilon }}_{\mathrm{cr}}\left(y,t\right)$, ${\hat{\varepsilon }}_{\mathrm{net}}\left(y,t\right)$, ${\hat{\varepsilon }}_{p\_\mathrm{el}}\left(t\right)$, ${\hat{\varepsilon }}_{p\_\mathrm{re}}\left(t\right)$ and ${\hat{\varepsilon }}_{p}\left(t\right)$ in eight governing equations from Equation (9) to Equation (16), a single-closed form governing equation with respect to $\hat{P}\left(t\right)$ is derived in Section 2.2.

### 2.2. Formulation

#### 2.2.1. Deriving Integro-Differential Equation for $\hat{P}\left(t\right)$

Integrating Equation (16) with respect to t results in

(17) $${\hat{\varepsilon }}_{p}\left(t\right)={\hat{\varepsilon }}_{\mathrm{net}}\left(-e,t\right)+C_{1}$$

where $C_{1}$ can be determined by using two initial conditions, ${\hat{\varepsilon }}_{p}\left(0\right)={\hat{\varepsilon }}_{p\_\mathrm{el}}\left(0\right)$ and ${\hat{\varepsilon }}_{\mathrm{net}}\left(-e,0\right)={\hat{\varepsilon }}_{\mathrm{el}}\left(-e,0\right)$, as follows:

(18) $$C_{1}={\hat{\varepsilon }}_{p}\left(0\right)-{\hat{\varepsilon }}_{\mathrm{net}}\left(-e,0\right)={\hat{\varepsilon }}_{p\_\mathrm{el}}\left(0\right)-{\hat{\varepsilon }}_{\mathrm{el}}\left(-e,0\right)$$

Substituting Equations (10) and (13) at y=−e and t=0 into Equation (18) yields

(19) $$C_{1}=\hat{P}\left(0\right)+{\hat{\varepsilon }}_{p\_d}\left(0\right)-\left\{-\Phi_{\mathrm{el}}\left(0\right)\gamma \left(-e\right)\hat{P}\left(0\right)-{\hat{\varepsilon }}_{p\_d}\left(0\right)\frac{-e}{e}\right\}=1+\Gamma$$

where $\hat{P}\left(0\right)=1$, $\Phi_{\mathrm{el}}\left(0\right)=1$ and $\Gamma =\gamma \left(-e\right)$.

Substituting Equation (13) into Equation (15) produces

(20) $$\hat{P}\left(t\right)={\hat{\varepsilon }}_{p}\left(t\right)-{\hat{\varepsilon }}_{p\_\mathrm{re}}\left(t\right)-{\hat{\varepsilon }}_{p\_d}\left(t\right)$$

Substituting Equation (17) with Equation (18) into Equation (20) results in

(21) $$\hat{P}\left(t\right)={\hat{\varepsilon }}_{\mathrm{net}}\left(-e,t\right)+1+\Gamma -{\hat{\varepsilon }}_{p\_\mathrm{re}}\left(t\right)-{\hat{\varepsilon }}_{p\_d}\left(t\right).$$

Substituting Equation (12) into Equation (21) produces

(22) $$\hat{P}\left(t\right)={\hat{\varepsilon }}_{\mathrm{el}}\left(-e,t\right)+{\hat{\varepsilon }}_{\mathrm{cr}}\left(-e,t\right)+{\hat{\varepsilon }}_{\mathrm{sh}}\left(t\right)+1+\Gamma -{\hat{\varepsilon }}_{p\_\mathrm{re}}\left(t\right)-{\hat{\varepsilon }}_{p\_d}\left(t\right)$$

${\hat{\varepsilon }}_{\mathrm{el}}\left(-e,t\right)$ in Equation (22) can be expressed as follows by using Equation (10) at y=−e:

(23) $${\hat{\varepsilon }}_{\mathrm{el}}\left(-e,t\right)=-\Phi_{\mathrm{el}}\left(t\right)\gamma \left(-e\right)\hat{P}\left(t\right)+{\hat{\varepsilon }}_{p\_d}\left(t\right)=-{\Gamma \Phi }_{\mathrm{el}}\left(t\right)\hat{P}\left(t\right)+{\hat{\varepsilon }}_{p\_d}\left(t\right).$$

Introducing a prestress transfer coefficient $\varphi \left(t\right)=\frac{1}{1+{\Gamma \Phi }_{\mathrm{el}}\left(t\right)}$ at time t, Equation (23) is rewritten as

(24) $${\hat{\varepsilon }}_{\mathrm{el}}\left(-e,t\right)=-\left\{\frac{1}{\varphi \left(t\right)}-1\right\}\hat{P}\left(t\right)+{\hat{\varepsilon }}_{p\_d}\left(t\right)$$

Substituting Equation (24) into Equation (22) results in the following equation:

(25) $$\hat{P}\left(t\right)=\frac{\varphi \left(t\right)}{\varphi \left(0\right)}+\varphi \left(t\right)\left\{{\hat{\varepsilon }}_{\mathrm{cr}}\left(-e,t\right)-{\hat{\varepsilon }}_{p\_\mathrm{re}}\left(t\right)+{\hat{\varepsilon }}_{\mathrm{sh}}\left(t\right)\right\}$$

where $\varphi \left(0\right)=\frac{1}{1+\Gamma }$.

${\hat{\varepsilon }}_{\mathrm{cr}}\left(-e,t\right)$ in Equation (25) can be expressed as follows by substituting Equation (9) into Equation (11) at y=−e

(26) $${\hat{\varepsilon }}_{\mathrm{cr}}\left(-e,t\right)=-\Gamma \left\{\int_{0}^{t}\Phi_{\mathrm{cr}}\left(t,\tau \right)\frac{\partial \hat{P}\left(\tau \right)}{\partial \tau }d\tau +\Phi_{\mathrm{cr}}\left(t,0\right)\right\}+\Phi_{\mathrm{cr}}\left(t,0\right){\hat{\sigma }}_{p\_d}.$$

Substituting Equation (13) into Equation (14), ${\hat{\varepsilon }}_{p\_\mathrm{re}}\left(t\right)$ in Equation (25) can be expressed as follows:

(27) $${\hat{\varepsilon }}_{p\_\mathrm{re}}\left(t\right)=\Phi_{\mathrm{re}}\left(t,0\right)+\Phi_{\mathrm{re}}\left(t,0\right){\hat{\varepsilon }}_{p\_d}\left(0\right).$$

Substituting Equations (26) and (27) into Equation (25) produces an integro-differential equation for $\hat{P}\left(t\right)$ as follows:

(28) $$\begin{array}{cc} \hat{P}\left(t\right)=\frac{\varphi \left(t\right)}{\varphi \left(0\right)} & +\varphi \left(t\right)[-\Gamma \left\{\int_{0}^{t}\Phi_{\mathrm{cr}}\left(t,\tau \right)\frac{\partial \hat{P}\left(\tau \right)}{\partial \tau }d\tau +\Phi_{\mathrm{cr}}\left(t,0\right)\right\}\\ & +\Phi_{\mathrm{cr}}\left(t,0\right){\hat{\sigma }}_{p\_d}-\Phi_{\mathrm{re}}\left(t,0\right)-\Phi_{\mathrm{re}}\left(t,0\right){\hat{\varepsilon }}_{p\_d}\left(0\right)+{\hat{\varepsilon }}_{\mathrm{sh}}\left(t\right)]. \end{array}$$

#### 2.2.2. Forward Finite Difference Equation

The numerical solution of Equation (28) for the dimensionless effective prestress force $\hat{P}\left(t\right)$ can be calculated by the finite difference method. First, the integral part in Equation (28) is rewritten in terms of $\frac{\partial \hat{P}\left(t\right)}{\partial t}$ as

(29) $$\int_{0}^{t}{\Gamma \Phi }_{\mathrm{cr}}\left(t,\tau \right)\frac{\partial \hat{P}\left(\hat{\tau }\right)}{\partial \tau }d\tau =\overset{n-1}{\underset{i=1}{\sum }}\int_{t_{i}}^{t_{i+1}}{\Gamma \Phi }_{\mathrm{cr}}\left(t,\tau \right)\frac{\partial \hat{P}\left(\tau \right)}{\partial \tau }d\tau$$

where $t_{1}=0$ and $t_{n}=t$.

Letting constant time increment $\Delta t=t_{i+1}-t_{i}=\frac{t}{n-1}$ for all i, the finite integrals from $t_{i}$ to $t_{i+1}$ in Equation (29) can be approximated through the trapezoidal rule as follows:

(30) $$\int_{t_{i}}^{t_{i+1}}{\Gamma \Phi }_{\mathrm{cr}}\left(t_{n},\tau \right)\frac{\partial \hat{P}\left(\tau \right)}{\partial \tau }d\tau \approx \frac{1}{2}\left\{\chi_{n,i}{\left.\frac{\partial \hat{P}\left(\tau \right)}{\partial \tau }\right|}_{\tau =t_{i}}+\chi_{n,i+1}{\left.\frac{\partial \hat{P}\left(\tau \right)}{\partial \tau }\right|}_{\tau =t_{i+1}}\right\}\Delta t$$

where $\chi_{n,i}={\Gamma \Phi }_{\mathrm{cr}}\left(t_{n},t_{i}\right)$.

The derivative $\frac{\partial \hat{P}\left(\tau \right)}{\partial \tau }$ in Equation (30) can be approximated through forward finite differences as

(31) $${\left.\frac{\partial \hat{P}\left(\tau \right)}{\partial \tau }\right|}_{\tau =t_{i}}\approx \frac{\hat{P}\left(t_{i+1}\right)-\hat{P}\left(t_{i}\right)}{\Delta t}.$$

Substituting Equation (31) into Equation (30) produces

(32) $$\begin{array}{c} \int_{t_{i}}^{t_{i+1}}{\Gamma \Phi }_{\mathrm{cr}}\left(t_{n},\tau \right)\frac{\partial \hat{P}\left(\tau \right)}{\partial \tau }d\tau \\ \;\;\;\;\approx \frac{1}{2}\left[\chi_{n,i+1}\hat{P}\left(t_{i+2}\right)+\left\{\chi_{n,i}-\chi_{n,i+1}\right\}\hat{P}\left(t_{i+1}\right)-\chi_{n,i}\hat{P}\left(t_{i}\right)\right]. \end{array}$$

Substituting Equation (29) incorporated with Equation (32) into Equation (28) yields the finite difference equation of Equation (28)

(33) $$\begin{array}{cc} \hat{P}\left(t_{n}\right)=\frac{\varphi \left(t_{n}\right)}{\varphi \left(t_{1}\right)} & +\varphi \left(t_{n}\right)[\Phi_{\mathrm{cr}}\left(t_{n},t_{1}\right)\left\{{\hat{\sigma }}_{p\_d}-\Gamma \right\}-\Phi_{\mathrm{re}}\left(t_{n},t_{1}\right)\left\{1+{\hat{\varepsilon }}_{p\_d}\left(t_{1}\right)\right\}\\ & +{\hat{\varepsilon }}_{\mathrm{sh}}\left(t_{n}\right)]-\varphi \left(t_{n}\right)\sum_{i=1}^{n-1}\psi_{n,i} \end{array}$$

where $\psi_{n,i}=\frac{1}{2}\left[\chi_{n,i+1}\hat{P}\left(t_{i+2}\right)+\left\{\chi_{n,i}-\chi_{n,i+1}\right\}\hat{P}\left(t_{i+1}\right)-\chi_{n,i}\hat{P}\left(t_{i}\right)\right]$.

The numerical procedures to solve Equation (33) for $\hat{P}\left(t_{n}\right)$ is provided in Appendix A.

### 2.3. PINN for Solving the Integro-Differential Governing Equation

#### 2.3.1. Approximate Integro-Differential Equation of Equation (28) for Using the PINN

The advantage of the PINN over conventional numerical methods lies in solving partial differential equations in strong forms without discretization through automatic differentiation. When the governing equation is expressed as an integro-differential equation like Equation (28), the PINN can be employed to solve it through automatic differentiation for integer-order derivatives and approximate integral operators based on Gaussian quadrature [37].

Substituting $\hat{Z}\left(t\right)=\frac{\hat{P}\left(t\right)}{\varphi \left(t\right)}$ into Equation (28) results in the integro-differential equation in terms of $\hat{Z}\left(t\right)$ as follows:

(34) $$\begin{array}{cc} \hat{Z}\left(t\right)=\frac{1}{\varphi \left(0\right)} & +[-\Gamma \{\int_{0}^{t}\Phi_{\mathrm{cr}}\left(t,\tau \right)\left\{\varphi \left(\tau \right)\frac{\partial \hat{Z}\left(\tau \right)}{\partial \tau }+\hat{Z}\left(\tau \right)\frac{\partial \varphi \left(\tau \right)}{\partial \tau }\right\}d\tau \\ & +\Phi_{\mathrm{cr}}\left(t,0\right)\}+\Phi_{\mathrm{cr}}\left(t,0\right){\hat{\sigma }}_{p\_d}-\Phi_{\mathrm{re}}\left(t,0\right)\\ & -\Phi_{\mathrm{re}}\left(t,0\right){\hat{\varepsilon }}_{p\_d}\left(0\right)+{\hat{\varepsilon }}_{\mathrm{sh}}\left(t\right)]. \end{array}$$

The right-hand side of Equation (34) can be rearranged by separating the integral term from the non-integral terms as follows:

(35) $$\begin{array}{l} \hat{Z}\left(t\right)\\ =\underset{\mathrm{RHS}1}{\underset{︸}{-\Gamma \int_{0}^{t}\Phi_{\mathrm{cr}}\left(t,\tau \right)\left\{\varphi \left(\tau \right)\frac{\partial \hat{Z}\left(\tau \right)}{\partial \tau }+\hat{Z}\left(\tau \right)\frac{\partial \varphi \left(\tau \right)}{\partial \tau }\right\}d\tau }}\\ +\underset{\mathrm{RHS}2}{\underset{︸}{\left[\frac{1}{\varphi \left(0\right)}-{\Gamma \Phi }_{\mathrm{cr}}\left(t,0\right)+\Phi_{\mathrm{cr}}\left(t,0\right){\hat{\sigma }}_{p\_d}-\Phi_{\mathrm{re}}\left(t,0\right)-\Phi_{\mathrm{re}}\left(t,0\right){\hat{\varepsilon }}_{p\_d}\left(0\right)+{\hat{\varepsilon }}_{\mathrm{sh}}\left(t\right)\right]}} \end{array}$$

where the integral term and non-integral terms are denoted as RHS1 and RHS2, respectively. Then, RHS1 is approximated through the Gauss quadrature:

(36) $$\begin{array}{c} \mathrm{RHS}1=-\Gamma \int_{0}^{t}\Phi_{\mathrm{cr}}\left(t,\tau \right)\left\{\varphi \left(\tau \right)\frac{\partial \hat{Z}\left(\tau \right)}{\partial \tau }+\hat{Z}\left(\tau \right)\frac{\partial \varphi \left(\tau \right)}{\partial \tau }\right\}d\tau \\ \;\;\;\;\;\;\approx -\Gamma \sum_{i=1}^{n_{\mathrm{gq}}}w_{i}\Phi_{\mathrm{cr}}\left(t,\tau_{i}\left(t\right)\right)\{\varphi \left(\tau \right)\frac{\partial \hat{Z}\left(\tau \right)}{\partial \tau }\\ \;\;\;\;\;\;+\hat{Z}\left(\tau \right)\frac{\partial \varphi \left(\tau \right)}{\partial \tau }\}{|}_{\tau =\tau_{i}\left(t\right)} \end{array}$$

where $n_{\mathrm{gq}}$, $w_{i}$, and $\tau_{i}\left(t\right)$ represent the number of Gauss quadrature points, weight factors, and Gauss points associated with the integral interval from 0 to t, respectively. Note that automatic differentiation is used to analytically derive ${\left.\frac{\partial \hat{P}\left(\tau \right)}{\partial \tau }\right|}_{\tau =\tau_{i}\left(t\right)}$ at each Gauss point. Substituting Equation (36) into Equation (35) yields the approximate integro-differential equation that is solved through the PINN. Once $\hat{Z}\left(t\right)$ is obtained by the PINN, $\hat{P}\left(t\right)$ can be calculated by multiplying $\varphi \left(t\right)$ to $\hat{Z}\left(t\right)$.

#### 2.3.2. Optimal Hyperparameter Combinations for the PINN to Solve Equation (34)

**Figure 2:**
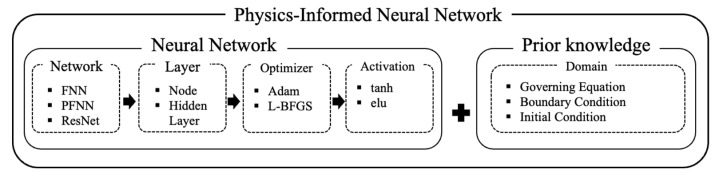
The basic architecture of the PINN.

The basic architecture of the PINN is illustrated in Figure 2. By incorporating prior knowledge directly into the domain of structured artificial neural networks with network structures, layer activation functions, and optimization functions, the PINN can be trained.

**Figure 3:**
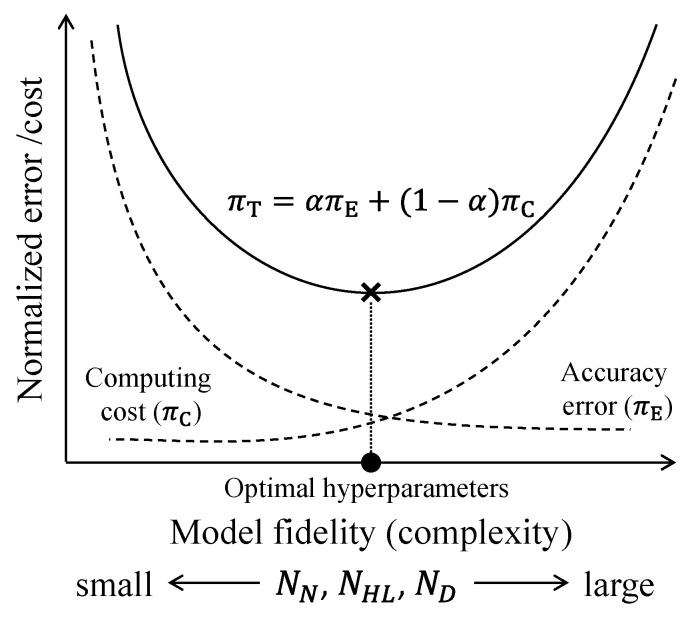
Schematic of determining optimal hyperparameters for the PINN using a trade-off curve ($\pi_{T}$) between accuracy error and computing cost where $\pi_{T}=\alpha \pi_{E}+\left(1-\alpha \right)\pi_{C}$ and 0≤α≤1.

The PINN architecture shown in Figure 2 has three primary hyperparameters: the number of nodes ($N_{N}$), the number of hidden layers ($N_{\mathrm{HL}}$) and the number of domains ($N_{D}$). Here, $N_{N}$ and $N_{\mathrm{HL}}$ account for the model complexity which enables capturing complicated physical behaviors embedded in the governing equation while $N_{D}$ accounts for the amount of prior information provided by the governing equation. Therefore, these three primary hyperparameters determine the model fidelity of the PINN. As these hyperparameters increase, the model fidelity of the PINN improves meaning that the PINN predicts the solution of the governing equation more accurately and consistently. In contrast, the computing cost is likely to decrease as the hyperparameters decrease. In this regard, considering the trade-off between the accuracy error and computing cost, optimal hyperparameter combinations for the PINN should be determined. Figure 3 illustrates a schematic of determining optimal hyperparameter combinations considering this trade-off. The accuracy error monotonically decreases with the primary hyperparameters while the computing cost monotonically increases. By introducing a weight factor α that balances the importance of the solution accuracy and the computing cost, optimal hyperparameters can be determined so that the trade-off curve $\pi_{T}$ is minimized.

## 3. Results and Discussion

### 3.1. Numerical Verification

#### 3.1.1. Rectangular Cross Section

**Figure 4:**
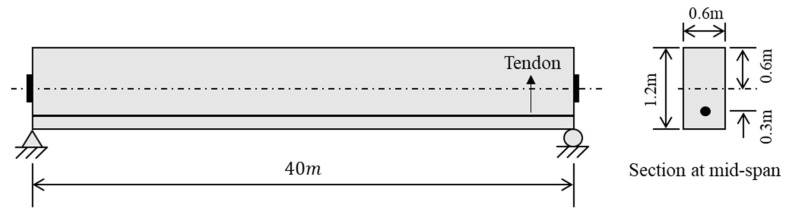
A 40 m long simply-supported PSC beam with a rectangular cross section.

Figure 4 presents a 40 m long simply-supported PSC beam with a rectangular cross-section (width 0.6 m × height 1.2 m). The specification of the PSC beam is provided in Table 1. Wet curing is conducted until $t_{s}=3\;\mathrm{days}$, after which shrinkage begins. The relative humidity of ambient environment RH = 70%. The PSC beam is post-tensioned to the initial prestressing force such that the initial prestressing ratio $f_{\mathrm{pi}}/f_{\mathrm{py}}=0.7$. It is assumed that the losses of prestressing force due to friction and anchorage draw-in have been taken into account in the initial prestressing ratio. The profile of the tendon is straight with a constant eccentricity ratio e/h=0.3. The time-dependent properties of concrete are calculated by using CEB-FIP Model Code 1990 [63]. The relaxation of the tendon is calculated by modifying the intrinsic stress relaxation function proposed by [64] for low-relaxation steel.

The forward finite difference method elaborated in Section 2.2.2 and Appendix A is adopted to obtain reference solutions of Equation (34). The analysis period is from $d_{0}=28\;\mathrm{days}$ on which the initial prestress transfer is carried out to 365 days, i.e., 0≤t≤337. Time increment Δt is 0.01685 day in the finite difference equation. The middle of the PSC beam is a section of interest for time-dependent analysis.

Table 2 presents hyperparameter combinations for varying $N_{N}$, $N_{\mathrm{HL}}$ and $N_{D}$ which are three primary hyperparameters described in Section 2.3.2. $N_{D}$ is also known as the number of residual points. The residual points are randomly selected at the beginning of training and remain static during the training process. Once the residual points are selected, the deep neural networks are optimized to satisfy the physics imposed by the integro-differential governing equation at the selected residual points. A total of 144 combinations of primary hyperparameters are considered. The ‘elu’ activation function is adopted for the activation function and the Adam optimizer followed by L-BFGS is employed for optimization. Equation (36) is accurately approximated using 40 Gauss quadrature points. All computing simulations were conducted on a workstation equipped with an Intel Core i9-10900X CPU and an NVIDIA Quadro RTX 6000 GPU, running a Windows 10 Pro operating system. The DeepXDE [37] was adopted to implement the PINNs with hyperparameter combinations for solving Equation (34) in Table 2.

**Figure 5:**
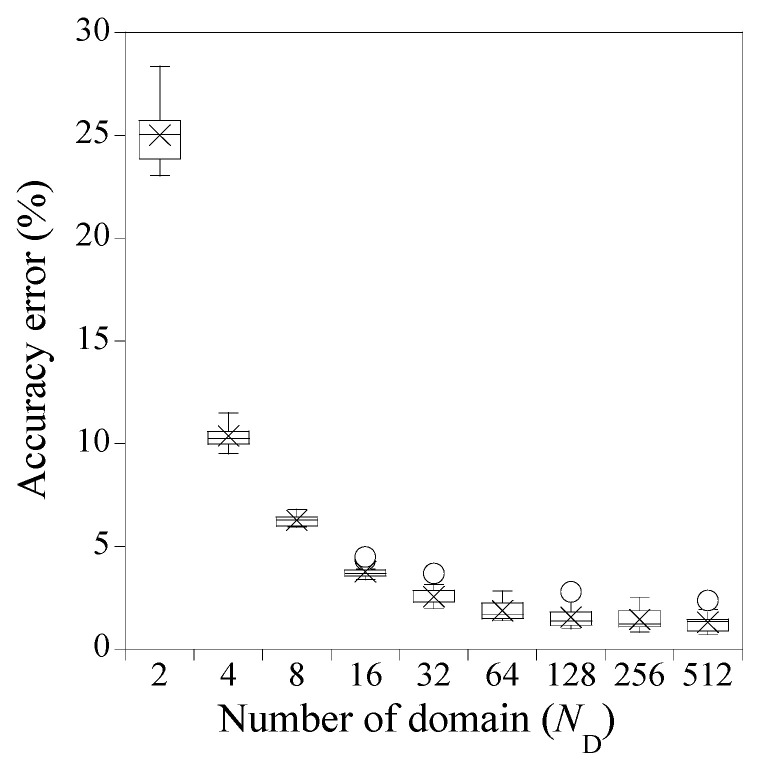
Accuracy error of PINN for solving Equation (34). ‘×’ and ‘o’ represent mean values and outliers, respectively.

For each hyperparameter combination outlined in Table 2, accuracy errors are calculated through 30 times Monte-Carlo simulations to confirm the consistency of the predicted solutions. Here, the accuracy error is defined as the relative root mean square error between the predicted solution and the reference solution calculated from Equation (33). Figure 5 presents a box plot that exhibits the 16 mean values of the 30 accuracy errors, each corresponding to 1 of the 16 hyperparameter combinations per number of domains. The symbols ‘×’ and ‘o’ represent the mean values and the outliers of 16 mean values of the 30 accuracy errors, respectively. The median values in the box plot align closely with the mean values despite some outliers.

**Figure 6:**
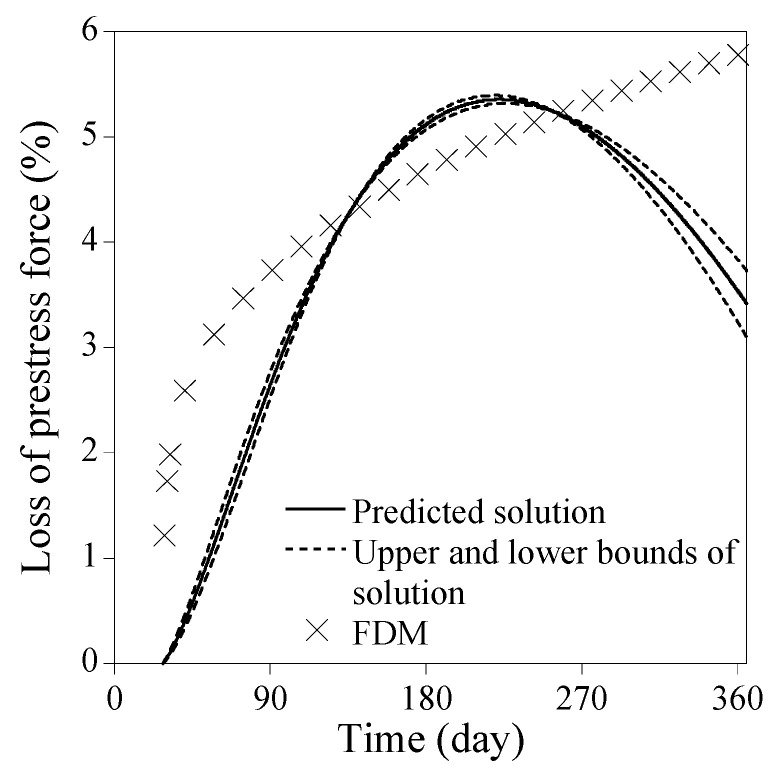
Poorly fitted results from PINN for solving Equation (34) [$N_{N}=128$, $N_{\mathrm{HL}}=7$, $N_{D}=2$].

**Figure 7:**
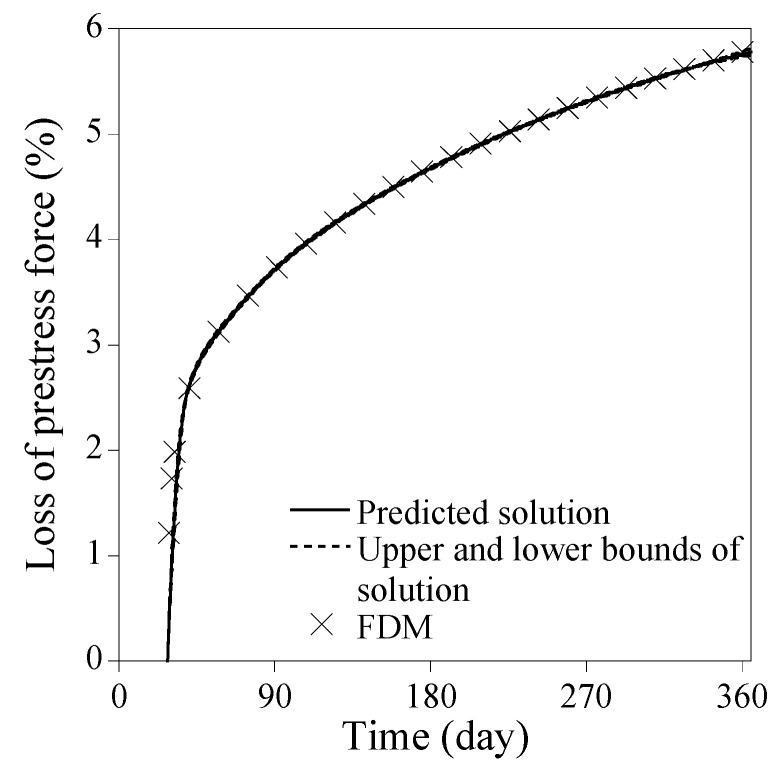
Well-fitted results from PINN for solving Equation (34) [$N_{N}=128$, $N_{\mathrm{HL}}=7$, $N_{D}=32$].

**Figure 8:**
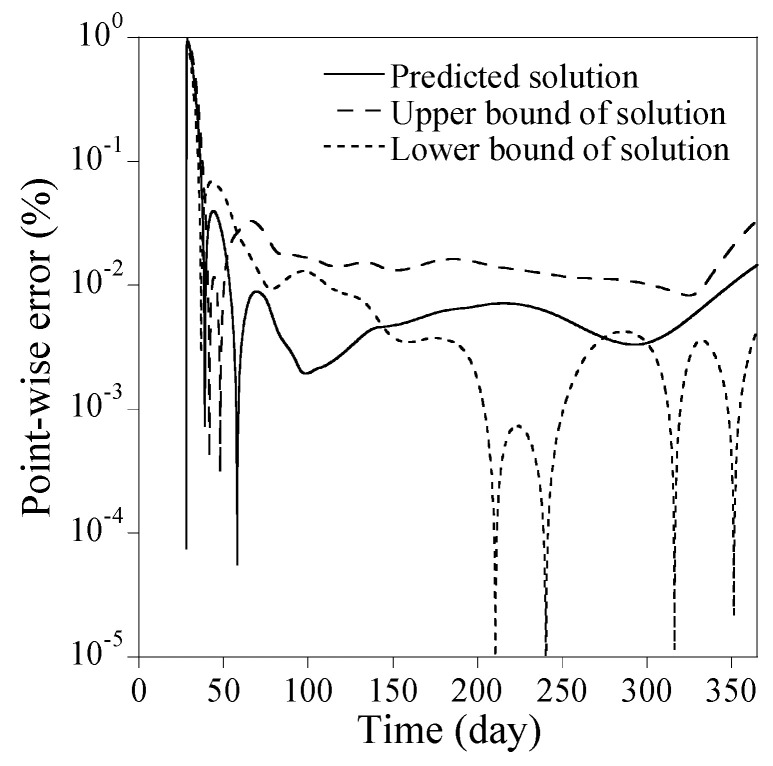
Point-wise errors of the well-fitted results from PINN in Figure 7.

Three implications can be drawn from Figure 5. Firstly, the most critical hyperparameter is $N_{D}$; the accuracy error decreases rapidly with $N_{D}<32$ and then slows down with $N_{D}\geq 32$. Secondly, when $N_{D}$ is small, the accuracy error deviates to some extent with varying $N_{N}$ and $N_{\mathrm{HL}}$. As $N_{D}$ increases, however, the impact of $N_{N}$ and $N_{\mathrm{HL}}$ on the accuracy error decreases quickly. Thirdly, when $N_{D}$ is very small, the predicted solution from PINN is likely to poorly fit the reference solution as shown in Figure 6. In contrast, when $N_{D}\geq 32$, the predicted solutions agree with the reference solution very well, as shown in Figure 7. The upper and lower bounds depicted in Figure 6 and Figure 7, which are set at a standard deviation away from the predicted solution, represent the solution precision for 30 times trials during the Monte-Carlo simulation. As observed in Figure 6, the solution precision is low when $N_{D}$ is small, regardless of large $N_{N}$ and $N_{\mathrm{HL}}$. Conversely, when $N_{D}\geq 32$, the solution accuracy improves significantly for the identical $N_{N}$ and $N_{\mathrm{HL}}$, as shown in Figure 7. The point-wise errors of the well-fitted results from PINN in Figure 7 are presented in Figure 8.

**Figure 9:**
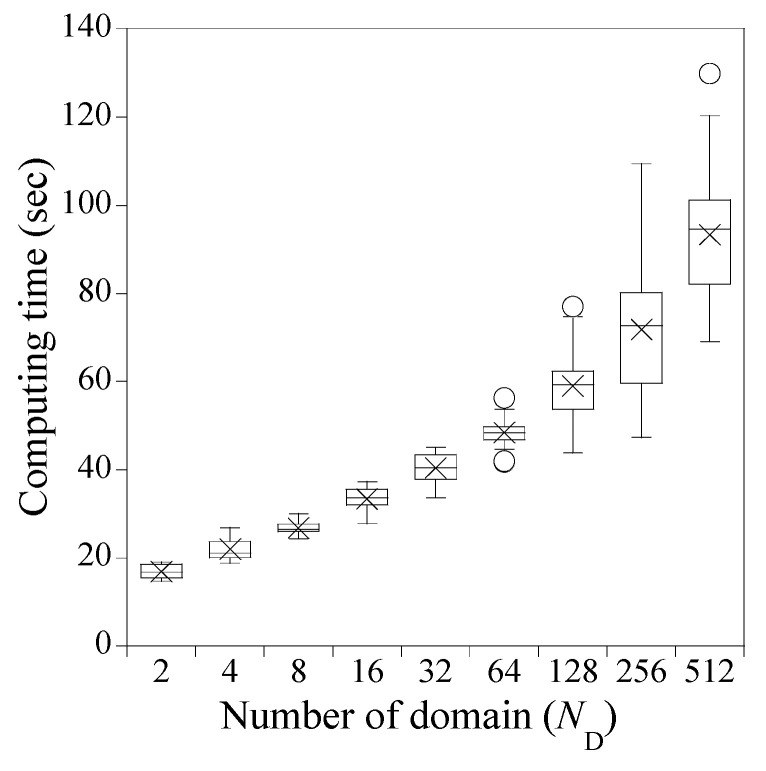
Computing time of PINN for solving Equation (34). ‘×’ and ‘o’ represent mean values and outliers, respectively.

Similar to Figure 5, Figure 9 illustrates a box plot that provides the mean values of the 30 computing times, each corresponding to 1 of the 16 hyperparameter combinations per number of domains. Similar to Figure 5, the median values in the box plot are almost identical to the mean values, though some outliers are observed. The computing time increases monotonically as $N_{D}$ increases. Up to $N_{D}=64$, the computation time remains constant regardless of $N_{N}$ and $N_{\mathrm{HL}}$, while for $N_{D}\geq 128$, the computation time increases with $N_{N}$ and $N_{\mathrm{HL}}$.

**Figure 10:**
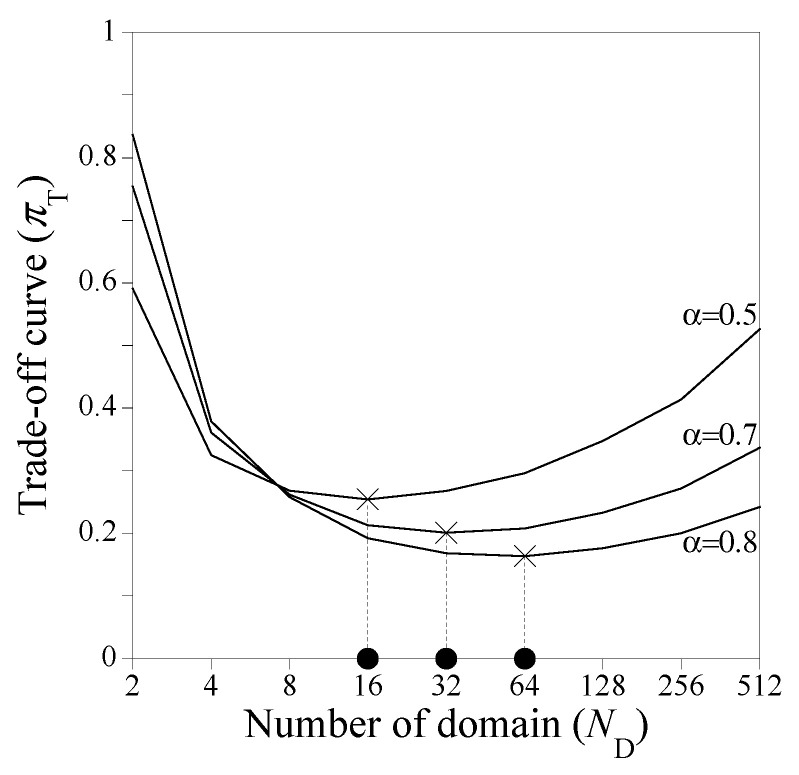
Determining optimal $N_{D}$ through trade-off curves for different α as described in Figure 3.

The optimal $N_{D}$ is determined by using the trade-off curve between accuracy error and computing cost in Figure 3. The accuracy error $\pi_{E}$ and the computing cost $\pi_{C}$ in Figure 3 are calculated with respect to $N_{D}$ by using the associated mean values in Figure 5 and Figure 9, respectively. Note that the mean values in Figure 5 and Figure 9 are normalized, respectively, so that the maximum values of $\pi_{E}$ and $\pi_{C}$ become unit values. Figure 10 illustrates trade-off curves to determine an optimal hyperparameter $N_{D}$ for three different weight factors α=0.5, 0.7 and  0.8. When α=0.5, the solution accuracy and the computing cost are equally important, while the solution accuracy is four times more important than the computing cost when α=0.8. Observing Figure 10, the optimal $N_{D}$ corresponds to 16, 32, and 64 for α=0.5, 0.7, and  0.8, respectively.

**Figure 11:**
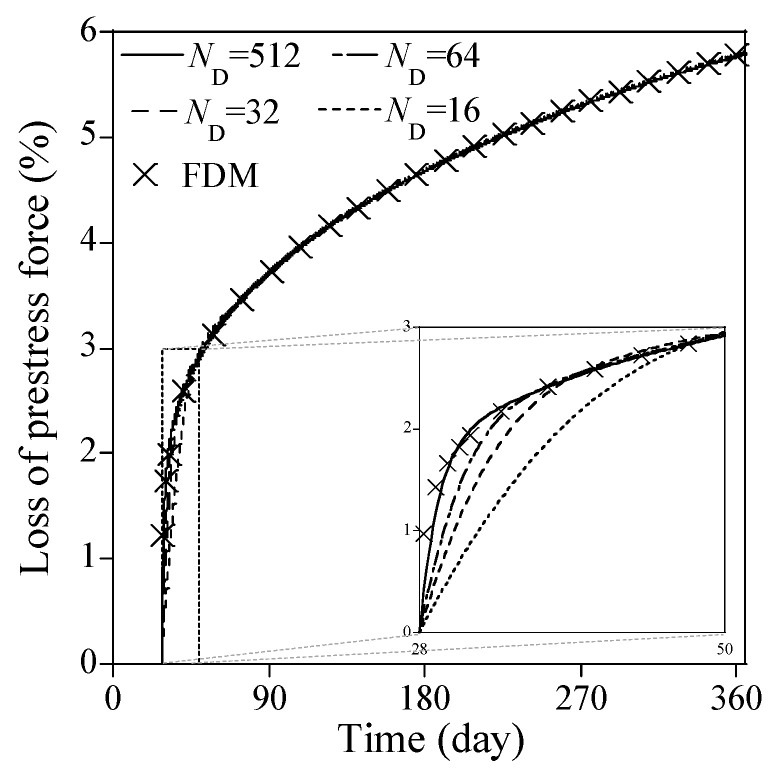
The predicted solutions using optimal $N_{D}$ for different α from Figure 10 with $N_{N}=64$ and $N_{\mathrm{HL}}=8$.

**Figure 12:**
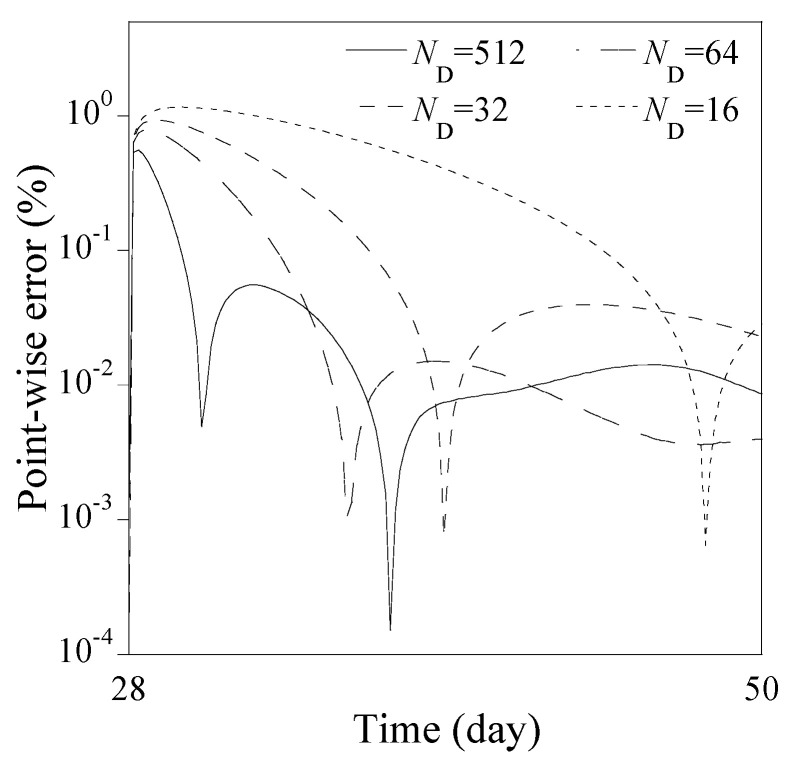
Point-wise errors of the predicted solutions from 28 days to 50 days as in Figure 11.

Figure 11 presents the predicted solutions from Equation (34) for $N_{D}=16$, 32, and 64, respectively, with $N_{N}=64$ and $N_{\mathrm{HL}}=8$. Note that each predicted solution is the mean of 30 solutions from the Monte-Carlo simulation for each combination of $N_{D}$, $N_{N}$ and $N_{\mathrm{HL}}$. For comparison purposes, the predicted solution corresponding to $N_{D}=512$, $N_{N}=128$ and $N_{\mathrm{HL}}=8$ is presented as standing for the most accurate solution available from the PINN, while the numerical solution from the finite difference method is provided as the reference solution. Overall, the three predicted solutions for $N_{D}=16$, 32, and 64 agree very well with that for $N_{D}=512$, and the reference solution after 60 days, though there exist slight differences from 28 days to 50 days. As $N_{D}$ increases, these differences vanish, as shown at the right bottom in Figure 11. The corresponding point-wise errors from 28 days to 50 days are presented in Figure 12.

Based on the observation of Figure 10 and Figure 11, the optimal combinations of the primary hyperparameters in the PINNs are determined as $N_{D}=32$, $N_{N}=64$ and $N_{\mathrm{HL}}=8$. Note that the number of nodes ($N_{N}$) and the number of hidden layers ($N_{\mathrm{HL}}$) affect neither the accuracy error nor the computation time when $N_{D}=$ 32. Therefore, this hyperparameter combination is adopted in PINNs to solve Equation (34) throughout the numerical verification otherwise mentioned hereafter.

**Figure 13:**
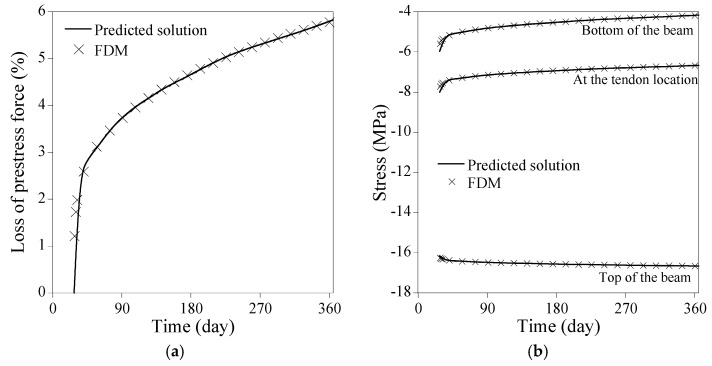
Comparison of numerical results from the PINNs ($N_{N}=64$, $N_{\mathrm{HL}}=8$, $N_{D}=32$) to the forward finite difference method: (**a**) loss of prestress force; (**b**) stress at the top and the bottom of the beam and at the tendon location.

**Figure 14:**
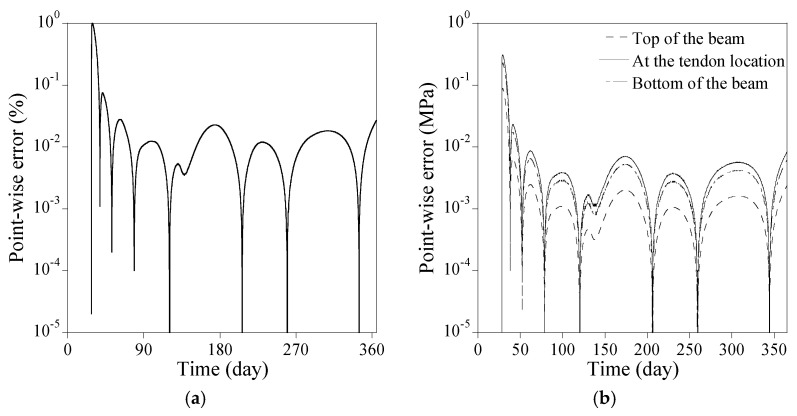
Point-wise error of the numerical results from the PINNs in Figure 13: (**a**) loss of prestress force; (**b**) stress at the top and the bottom of the beam and at the tendon location.

Figure 13a shows the comparison of the numerical results from the proposed PINN to the forward finite difference method regarding the loss of prestress force in case all time-dependent factors of the PSC beam are accounted for. The full coupling among the elastic modulus, creep and shrinkage of concrete, and the relaxation of tendon affects loss of prestress force with time. The loss of prestress force surges to 3.7% at 90 days and approaches 5.8% at 365 days. Figure 13b compares the stresses at the top and the bottom of the beam and at the tendon location. The final fractional magnitudes of the stresses at the top and the bottom of the beam, and at the tendon location become 103%, 83.3%, and 69.8%, respectively, compared to those on 28 days. The point-wise errors of the numerical results from the PINNs in Figure 13 are presented in Figure 14. The proposed method yields satisfactory results compared to the forward finite difference method. The absolute difference at 365 days in loss of prestress force is 0.027%, while that in all stresses at the top and the bottom of the beam and the tendon location is less than 0.20%.

#### 3.1.2. Conventional PSC I-Beam Section

**Figure 15:**
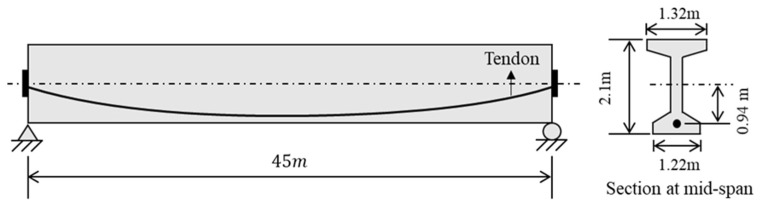
A 45 m long simply-supported PSC beam with an I-shaped cross section.

Figure 15 presents a 45 m long simply-supported PSC beam with an I-shaped cross section. The specification of the PSC beam is provided in Table 3. All design parameters not present in Table 3 are identical to those in Section 3.1.1 except for the tendon profile, which is parabolic with a maximum eccentricity ratio e/h=0.445 at the middle of the beam.

The analysis of time-dependent behaviors of the PSC beam is conducted by using the forward finite difference method in the same way described in Section 3.1.1 to obtain the reference solution. The analysis period is from the $d_{0}=7\;\mathrm{days}$ on which the initial prestress transfer is conducted to 365 days, i.e., 0≤t≤358. The time increment Δt is 0.01685 days in the finite difference equation. The middle of the PSC beam is a section of interest for time-dependent analysis. The optimal combination of the primary hyperparameters in the PINNs is identical to that used in Section 3.1.1: $N_{N}=64$, $N_{\mathrm{HL}}=8$, $N_{D}=32$.

**Figure 16:**
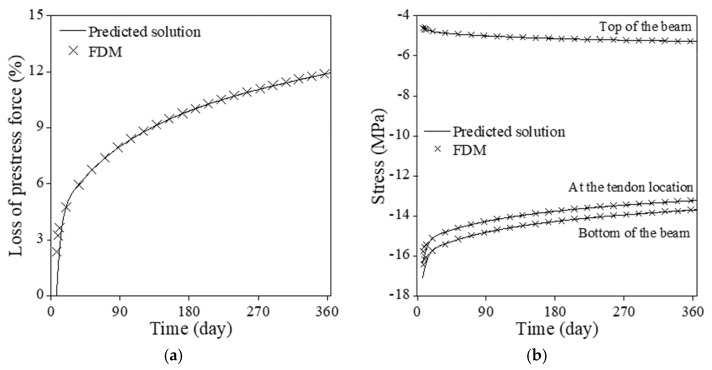
Comparison of numerical results from PINNs ($N_{N}=64$, $N_{\mathrm{HL}}=8$, $N_{D}=32$) to the forward finite difference method: (**a**) loss of prestress force; (**b**) stress at the top and the bottom of the beam and at the tendon location.

**Figure 17:**
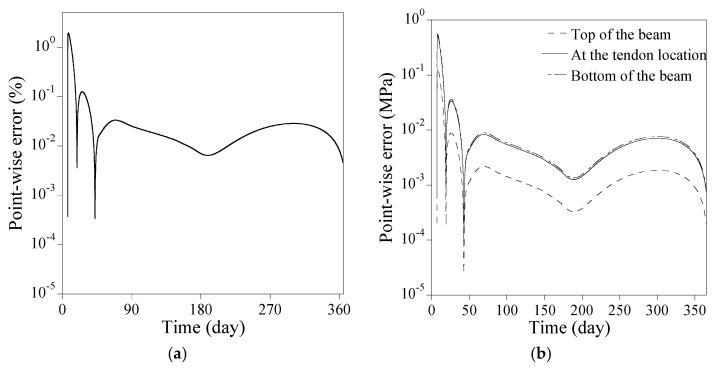
Point-wise error of the numerical results from the PINNs in Figure 16: (**a**) loss of prestress force; (**b**) stress at the top and the bottom of the beam and at the tendon location.

Figure 16a illustrates the comparison between the proposed PINNs and the forward finite difference method for loss of prestress force considering all time-dependent factors of the PSC beam. The complete interaction among the elastic modulus, concrete creep and shrinkage, and tendon relaxation influences the prestress loss over time. The loss of prestress force surges to 8.0% at 90 days and reaches 12% at 365 days. Figure 16b contrasts the stress levels at the beam’s top, bottom, and tendon locations. The final fractional stress magnitudes at these locations are 119%, 80.6%, and 80.0%, respectively, compared to those on 28 days. The point-wise errors of the numerical results from the PINNs in Figure 16 are presented in Figure 17. The proposed method yields satisfactory outcomes when compared to the forward finite difference method. The absolute difference at 365 days in prestress force loss is 0.042% while that in all stresses at the top and the bottom of the beam and the tendon location remains below 0.0075%.

## 4. Conclusions

In this study, a PINN approach was proposed to predict the early-age time-dependent behaviors of the PSC beam. The original contributions of this study are presented below:

- The dimensionless integro-differential governing equation is derived for the PINN. The governing equation accounts for time-dependent coupling among the effective prestress force and several factors including concrete creep and shrinkage, tendon relaxation, and changes in concrete elastic modulus.
- The fidelity of the PINN is determined by three primary hyperparameters: the number of nodes, the number of hidden layers, and the number of domains. Among these primary hyperparameters, the number of domains is the most critical factor for the accuracy and consistency of predicted solutions from the PINN. Optimal hyperparameter combinations for the PINN were determined by considering the trade-off between solution accuracy and computation time.
- The numerical results from the PINN with an optimal hyperparameter combination yielded satisfactory results, with absolute differences at 365 days in loss of prestress force being 0.027% and in all stresses at the top and bottom of the beam and the tendon location being less than 0.2% for PSC beams with a rectangular and an I-shaped section.
- The proposed PINN approach can effectively predict time-dependent behaviors of PSC beams, offering a promising alternative to conventional numerical methods. The proposed PINN can address the limitations of conventional machine learning by training deep neural networks that satisfy the governing equation associated with the complicated time-dependent behaviors of the PSC beam.

Future work will pursue a PINN to solve inverse problems such as estimating time-dependent material properties related to the concrete creep and shrinkage, and the relaxation of the tendon by using a limited amount of actual measurement data from the PSC beam. It is necessary to implement a more advanced PINN to solve the proposed integro-differential equation to improve the performance of the conventional PINN used in the present study. Future work will explore the incorporation of optimal adaptive activation functions [43,44,45,46], which could further improve its accuracy and convergence.

## Figures and Tables

**Table 1:** Specification of the PSC beam in Figure 4.

Material Properties	Specification
$E_{\mathrm{ci}}$	33,600 MPa
$E_{p}$	200 GPa
$A_{c}$	0.72 m^2^
$I_{c}$	0.0864 m^4^
$A_{p}/A_{c}$	1%
e/h	0.3
$f_{\mathrm{ck}}$	32 MPa
$f_{\mathrm{cu}}$	36 MPa
$f_{\mathrm{py}}$	1580 MPa
$f_{\mathrm{pi}}/f_{\mathrm{py}}$	0.7
$w_{c}$	25 kN/m^3^

**Table 2:** Hyperparameter combinations of the PINN for solving Equation (34).

Hyperparameter	Combinations
$N_{N}$	16, 32, 64, 128
$N_{\mathrm{NL}}$	5, 6, 7, 8
$N_{D}$	2, 4, 8, 16, 32, 64, 128, 256, 512
Activation function	elu
Optimizer	Adam + L-BFGS
Learning rate	0.001
Maximum iteration	10,000 for Adam optimizer
Number of Gauss quadrature points	40

**Table 3:** Specification of the PSC I-beam in Figure 15.

Material Properties	Specification
$E_{\mathrm{ci}}$	35,385 MPa
$E_{p}$	200 GPa
$A_{c}$	1.084 m^2^
$I_{c}$	0.6454 m^4^
$A_{p}/A_{c}$	0.972%
e/h	0.445
$f_{\mathrm{ck}}$	40 MPa
$f_{\mathrm{cu}}$	44 MPa
$f_{\mathrm{py}}$	1580 MPa
$f_{\mathrm{pi}}/f_{\mathrm{py}}$	0.7
$w_{c}$	25 kN/m^3^

## Data Availability

Data available on request from the corresponding author.

## Notations

t	Time (day) beginning from the initial prestress transfer
$d_{0}$	The age of concrete at t=0
$E_{c}\left(t\right)$	Elastic modulus of concrete at time t
$E_{c0}$	Elastic modulus of concrete at t=0 (i.e., $E_{c}\left(0\right))$
$E_{\mathrm{ci}}$	Elastic modulus of concrete at the age of 28 days (i.e., $E_{c}\left(28-d_{0}\right))$
$E_{p}$	Elastic modulus of the tendon
$n\left(t\right)$	Modular ratio at time t (i.e., $E_{p}/E_{c}\left(t\right)$)
$A_{p}$	Gross section area of tendons
$A_{c}$	Net section area of concrete of PSC beam
y	Vertical coordinate with the origin at the centroid of the net concrete section of the beam
h	Height of the PSC beam
e	Vertical distance from the centroid of the net concrete section of the beam
$f_{\mathrm{ck}}$	Characteristic compressive strength of concrete
$f_{\mathrm{cu}}$	Characteristic ultimate strength of concrete
$f_{\mathrm{py}}$	Yield stress of the tendon
$f_{\mathrm{pi}}$	Initial stress of the tendon
$w_{c}$	Specific weight of concrete per unit volume
$P_{i}$	Initial effective prestress force of tendon after elastic shortening of concrete at t=0
$P\left(t\right)$	Effective prestress force of tendon at time t
$M_{d}$	Bending moment induced by the self-weight of the beam
$\sigma \left(y,t\right)$	Axial-bending stress at location y and time t
$\varepsilon_{\mathrm{el}}\left(y,t\right)$	Elastic strain of concrete at location y and time t
$\varepsilon_{\mathrm{cr}}\left(y,t\right)$	Creep strain of concrete at location y and time t
$\varepsilon_{\mathrm{sh}}\left(t\right)$	Shrinkage strain of concrete at time t.
$\varepsilon_{\mathrm{net}}\left(y,t\right)$	Net strain of concrete at location y and time t
$\varepsilon_{p}\left(t\right)$	Net strain of tendon at time t
$\varepsilon_{p_{\mathrm{el}}}\left(t\right)$	Elastic strain of tendon at time t
$\varepsilon_{p_{\mathrm{re}}}\left(t\right)$	Relaxed strain of tendon at time t
${\tilde{\Phi }}_{\mathrm{cr}}\left(t,\tau \right)$	Creep coefficient at time t for the unit strain of which the corresponding stress is $E_{\mathrm{ci}}$ applied at time τ
$\Phi_{\mathrm{cr}}\left(t,\tau \right)$	Creep coefficient at time t for unit strain of which the corresponding stress is $E_{c0}$ applied at time τ (i.e., $\frac{E_{c0}}{E_{\mathrm{ci}}}{\tilde{\Phi }}_{\mathrm{cr}}\left(t,\tau \right)$)
$\Phi_{\mathrm{re}}\left(t,\tau \right)$	Relaxation function of the tendon at time t
$\Phi_{\mathrm{el}}\left(t\right)$	The ratio of $E_{c0}$ to $E_{c}\left(t\right)$ at time t (i.e., $\frac{E_{c0}}{E_{c}\left(t\right)})$
$\varphi \left(t\right)$	Prestress transfer coefficient at time t, i.e., $\frac{1}{1+n\left(t\right)A_{p}\left(\frac{1}{A_{c}}+\frac{e^{2}}{I_{c}}\right)}$ or $\frac{1}{1+{\Gamma \Phi }_{\mathrm{el}}\left(t\right)}$
$\gamma \left(y\right)$	$n\left(0\right)A_{p}\left(\frac{1}{A_{c}}-\frac{e}{I_{c}}y\right)$
Γ	$\gamma \left(-e\right)=n\left(0\right)A_{p}\left(\frac{1}{A_{c}}+\frac{e^{2}}{I_{c}}\right)$
$\varepsilon_{p\_d}\left(t\right)$	Elastic strain of concrete at the location of the tendon due to self-weight of the beam at time t (i.e., $\frac{\Phi_{\mathrm{el}}\left(t\right)}{E_{c0}}\sigma_{p\_d}\left(t\right)=\Phi_{\mathrm{el}}\left(t\right)\frac{M_{d}}{E_{c0}I_{c}}e$)
	Symbol for defining the associated dimensionless variables normalized by $P_{i}$, $\frac{P_{i}}{E_{p}A_{p}}$ and $E_{c0}\left(\frac{P_{i}}{E_{p}A_{p}}\right)$ for forces, strains, and stresses, respectively.

## Appendix A. Numerical Procedures to Calculate $\hat{P}\left(t_{n}\right)$ from the Finite Difference Equation (33)

(1) For $t_{n}=t_{1}=0$, Equation (33) is expressed for $\hat{P}\left(t_{1}\right)$ as follows:
  (A1) $$\begin{array}{cc} \hat{P}\left(t_{1}\right)=\frac{\varphi \left(t_{1}\right)}{\varphi \left(t_{1}\right)} & +\varphi \left(t_{1}\right)[\underset{0}{\underset{︸}{\Phi_{\mathrm{cr}}\left(t_{1},t_{1}\right)}}\left({\hat{\sigma }}_{p\_d}-\Gamma \right)-\underset{0}{\underset{︸}{\Phi_{\mathrm{re}}\left(t_{1},t_{1}\right)}}\left\{1+{\hat{\varepsilon }}_{p\_d}\left(t_{1}\right)\right\}\\ & +\underset{0}{\underset{︸}{{\hat{\varepsilon }}_{\mathrm{sh}}\left(t_{1}\right)}}]=1 \end{array}$$

Note that $\hat{P}\left(t_{1}\right)$ in Equation (A1) is identical to the initial condition of the dimensionless effective prestress force, i.e., $\hat{P}\left(0\right)=1$.

(2) For $t_{n}=t_{2}=\Delta t$, Equation (33) is written for $\hat{P}\left(t_{2}\right)$ as
  (A2) $$\begin{array}{c} \hat{P}\left(t_{2}\right)=\frac{\varphi \left(t_{2}\right)}{1+\frac{1}{2}\varphi \left(t_{2}\right)\chi_{2,1}}\{\frac{1}{\varphi \left(t_{1}\right)}+\left[\phi_{2,1}\left({\hat{\sigma }}_{p\_d}-\Gamma \right)-\rho_{2,1}\left\{1+{\hat{\varepsilon }}_{p\_d}\left(t_{1}\right)\right\}+{\hat{\varepsilon }}_{\mathrm{sh}}\left(t_{2}\right)\right]\\ \;\;\;\;\;+\frac{1}{2}\chi_{2,1}\hat{P}\left(t_{1}\right)\} \end{array}$$
(3) $t_{n}\geq t_{3}=2\Delta t$, Equation (33) is expressed for $\hat{P}\left(t_{n}\right)$ as follows
  (A3) $$\begin{array}{cc} \hat{P}\left(t_{n}\right) & =\frac{\varphi \left(t_{n}\right)}{1+\varphi \left(t_{n}\right)\chi_{n,n-1}}\{\frac{1}{\varphi \left(t_{1}\right)}\\ & +\left[\phi_{n,1}\left({\hat{\sigma }}_{p\_d}-\Gamma \right)-\rho_{n,1}\left\{1+{\hat{\varepsilon }}_{p\_d}\left(t_{1}\right)\right\}+{\hat{\varepsilon }}_{\mathrm{sh}}\left(t_{n}\right)\right]\\ & -\frac{1}{2}\left[\left(\chi_{n,n-2}-2\chi_{n,n-1}\right)\hat{P}\left(t_{n-1}\right)-\chi_{n,n-2}\hat{P}\left(t_{n-2}\right)\right]\\ & -\sum_{i=1}^{n-3}\psi_{n,i}\} \end{array}$$
